# The Synthesis and Antitumor Activity of 1,8-Naphthalimide Derivatives Linked 1,2,3-Triazole

**DOI:** 10.3389/fbioe.2021.662432

**Published:** 2021-04-13

**Authors:** Zhong-jie Xu, Ying-jie Zhou, Jia-hao Wang, Long-fei Mao, Wei Li, Gui-qing Xu

**Affiliations:** ^1^College of Life Science and Technology, Xinxiang Medical University, Xinxiang, China; ^2^Henan Engineering Research Center of Chiral Hydroxyl Pharmaceutical, School of Chemistry and Chemical Engineering, Henan Normal University, Xinxiang, China

**Keywords:** acenaphthylene, synthesis, 1,8-naphthalimide-1,2,3-triazole derivatives, anti-proliferative activities, H1975

## Abstract

In this study, acenaphthylene was used as the raw material, and a series of novel 1,8-naphthalimide-1,2,3-triazole derivatives was obtained through oxidation, acylation, alkylation, and click reactions, and subsequently, their anti-tumor activities were tested. After screening, we found that Compound **5e** showed good activity against H1975 lung cancer cells, with the half maximal inhibitory concentration (IC_50_) reaching 16.56 μM.

## Introduction

**FIGURE 1:**
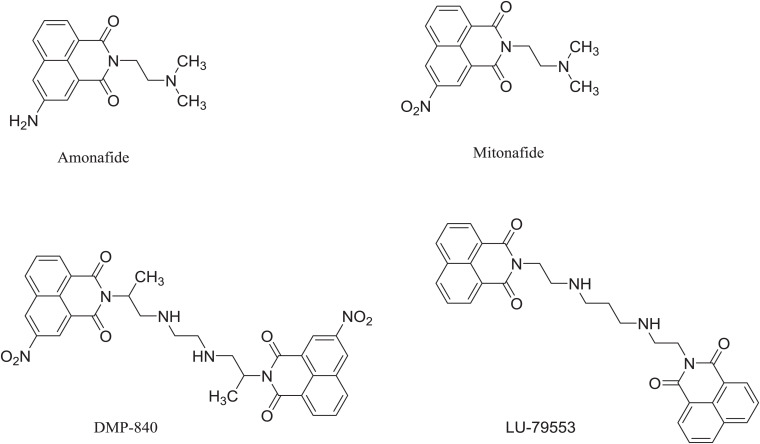
The reported naphthalimide derivatives.

Cancer is the leading cause of death worldwide. At present, surgery is still the first choice of treatment for many cancers, but easy recurrence and metastasis after surgery greatly affect the efficacy and prognosis (Gaitanis et al., 2018). For patients with local advanced tumors or distant metastases that are not suitable for surgery, traditional chemotherapy and radiotherapy show poor efficacy, and finding active and effective cancer treatments has become a main focus for researchers, especially the development of safe anti-cancer drugs. Based on DNA intercalators, small molecule drugs are being developed as anti-tumor drugs. Due to differences between the DNA of cancerous cells and normal cells, DNA intercalators play a significant role in treating tumors (Jiang, 2013). Naphthalimide derivatives have a special rigid planar structure, which gives them a strong ability to intercalate into DNA (Finney, 2006; Takahashi et al., 2007), so they have attracted extensive attention in the field of anti-tumor drug research and development. Some mononaphthalimides, such as amonafide (Wang et al., 2017; Johnson et al., 2019) and mitonafide (Sinha et al., 1985; Rosell et al., 1992), and bisnaphthalimides have entered the clinical research stage. Amonafide and mitonafide can not only intercalate into DNA and inhibit the synthesis of DNA and RNA, but also inhibit the activity of topoisomerase II, thereby inhibiting tumor cell division (Nazari and Ghandi, 2015). In addition, bisnaphthalimide compounds can bis-intercalate into DNA, have specificity to G-C bases, and exhibit stronger DNA binding and greater cytotoxicity than mononaphthalimides (Laquindanum et al., 1996; Wang and Yu, 2003; Langhals, 2004; Li and Wonneberger, 2012; Verma et al., 2013). For example, DMP-840 is a novel DNA-interactive chemical entity with excellent activityagainst human tumor colony-formingunits and its IC50 to Leukemia and other cell lines is 2.3–53 nM (Kirshenbaum et al., 1994; Nitiss et al., 1998). LU-79553, a bisnaphthalimides, exhibited dramatic antitumor activity in a variety of xenograft models and activity over a broad spectrum of tumor types made it an excellent candidate (Bousquet et al., 1995). For example, IC50 of LU-79553 is 18.0 μM to ovarian cell (Braña et al., 2004; Gonzalez-Bulnes and Gallego, 2009; Ferri et al., 2011; González-Bulnes and Gallego, 2012). Yet, the activity of naphthalimide derivatives on lung cancer is rarely reported (Figure 1).

**SCHEME 1:**
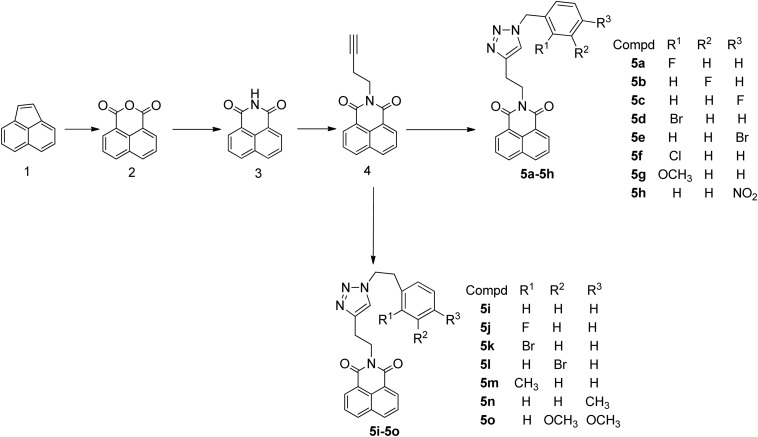
Reaction routes for the production of the target compounds.

Herein, a series of novel 1,8-naphthalimide-linked 1,2,3-triazole compounds were synthesized and tested for their antilung-cancer activity. From the raw material acenaphthylene (Compound **1**), 1,8-naphthalic anhydride (Compound **2**) was obtained by an oxidation reaction, and then the intermediate (Compound **3**) was obtained by an acylation reaction. Compound **3** underwent alkylation with 4-bromo-1-butyne to yield Compound **4**, which underwent a 1,3-dipolar cycloaddition reaction with different substituted azides to obtain target Compound **5** with a 1,2,3-triazole structure. The structures of the synthesized compounds Scheme 1) were confirmed by nuclear magnetic resonance (^1^H NMR and ^13^C NMR).

## Results and Discussion

### Effect of the Amount of Potassium Dichromate on the Yield of 1,8-Naphthalic Anhydride (Compound 2)

The key to this reaction is to use a strong oxidant to oxidize the carbon–carbon double bond to yield acid anhydride. We chose sodium dichromate as the oxidant and screened the amount of added sodium dichromate (Table 1). It can be seen from the table that under the condition that the reaction conditions remained unchanged, the reaction yield gradually increased with increasing amount of sodium dichromate. When the molar ratio of compound **1** to sodium dichromate is 1:2, the yield reached 80%. With the increase of the oxidant, the yield did not increase obviously. Therefore, the ratio of 1:2 was chosen as the optimal ratio to prepare compound **2**.

**TABLE 1:** Ratio optimization of sodiumdichromate to yield compound **2**.

Number	n (Compound 1):n (sodium dichromate)	Temperature (°C)	Time (h)	Yield (%)
1	1:1.0	80	6	59
2	1:1.2	80	6	63
3	1:1.4	80	6	69
4	1:1.6	80	6	74
5	1:1.8	80	6	78
6	1:2.0	80	6	80
7	1:2.2	80	6	77
8	1:2.4	80	6	81
9	1:2.6	80	6	79

### Influence of Reaction Temperature on the Yield of 1,8-Naphthalimide (Compound 3)

This reaction is an acylation reaction, and its temperature has a great influence on the yield of the reaction product. If the reaction is not complete, the raw materials will precipitate with the product during the cooling and crystallization process, which will affect the purity of the product. Therefore, we maintained the other reaction conditions unchanged and studied the reaction temperature (Table 2). With the increase of temperature, the yield increased. But the yield remained unchanged with the increase of temperature until 70°C. So we chose 70°C as the optimal reaction temperature.

**TABLE 2:** Optimization of the temperature of reaction to produce compound **3**.

Number	Temperature (°C)	Time (h)	Yield (%)
1	30	1.5	32
2	40	1.5	39
3	50	1.5	56
4	60	1.5	78
5	70	1.5	88
6	80	1.5	86
7	90	1.5	84
8	100	1.5	87

### Inhibitory Activity of Compounds on Tumor Cells

The inhibitory effect of Compounds **5a**–**5o** on H1975 lung cancer cells is shown in Table 3. Most of the compounds in this series had poor inhibitory effects on this cell type. Only compounds **5e**, **5g**, **5h**, and **5j** had a certain activity. Among them, **5e** showed the strongest effect with an IC_50_ of 16.56 μM. We then studied the structure-activity relationship. By comparing compounds **5a** and **5j**, **5c** and **5f**, **5k** and **5o**, and **5e** and **5m**, we clearly found that compounds with substituents at the *ortho* position of the benzene ring were more active than those with substituents at the *para* position. For compounds **5j** and **5k**, and **5h** and **5m**, we found that the activity of 1,2,3-triazole linked with the phenyl structure was generally stronger than those with 1,2,3-triazole linked with the benzyl structure. The results of **5e** and **5h** showed that when an electron withdrawing group with steric hindrance is at the *para* position of the benzene ring, the activity was obviously improved. The reason is worthy of further exploring.

**TABLE 3:** Data of the inhibitory effect of compounds **5a**–**5o** on lung cancer cell line.

Compound	IC_50_, μ M	Compound	IC_50_, μ M
**5a**	169.3 ± 1.38	**5i**	123 ± 1.24
**5b**	151.4 ± 1.35	**5j**	89.15 ± 1.27
**5c**	>200	**5k**	190.4 ± 1.47
**5d**	>200	**5l**	102.1 ± 1.26
**5e**	**16.56** ± **1.14**	**5m**	121.6 ± 1.21
**5f**	172.9 ± 1.47	**5n**	137.2 ± 1.30
**5g**	94.16 ± 1.47	**5o**	>200
**5h**	80.63 ± 1.27		

*The bold value indicates that the activity of the compound 5e is the best.*

## Conclusion

In summary, we designed and synthesized a series of novel 1,8-naphthalimide-1,2,3-triazole derivatives through simple and efficient methods, conducted anti-tumor activity studies on them, and found that compound **5e** had a good inhibitory effect on H1975 lung cancer cells. The results showed that compounds **5e** deserves further research about its anti-tumor activity and mechanism of action in order to find better compounds. Meanwhile, because the introduced substituents of our compounds are lipophilic, the solubility in water of our compounds is less than DMP. Our next step is to improve the water solubility of **5e**.

## Materials and Methods

1,8-naphthalimide-1,2,3-triazole derivatives were in-house synthesized. All reagents were obtained from commercial sources and used without further purification. All reactions were monitored by thin-layer chromatography (TLC). ^1^H and ^13^C spectra were recorded on a Bruker Avance 400 or 600 MHz spectrometer, respectively. NMR spectra were recorded in CDCl_3_ or DMSO-d_6_ at room temperature (20 ± 2 °C). ^1^H and ^13^C chemical shifts are quoted in parts per million downfield from TMS. **H1975** lung cancer cell line, DMEM medium and fetal bovine serum were purchased from ATCC (Virginia, United States). MTT powder and Dimethyl sulfoxide (DMSO) was purchased from Acros Organics (Morris Plains, NJ, United States).

### General Procedure for the Compound 2


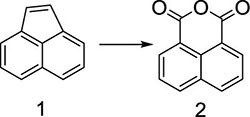


In a reaction flask, acenaphthylene (Compound **1**, 15 g, 0.1 mol) was added into 500 mL of glacial acetic acid, followed by adding 55 g (0.2 mol) of sodium dichromate. The mixture was stirred evenly at room temperature, and then the temperature was slowly increased to 80 °C, after which the reaction was carried out for 6 h. Thin layer chromatography (TLC) was used to monitor the progress of the reaction. When the reaction was complete, the reaction mixture was poured into 2,000 mL of ice water while it was hot. Solid precipitates appeared, and the reaction mixture was filtered, and the filter cake was dried to obtain 1,8-naphthalic anhydride (Compound **2**, 16 g), and the yield was 80%; ^1^H NMR (400 MHz, DMSO-*d*_6_): δ 8.55 (dd, *J*_1_ = 8.0 Hz, *J*_1_ = 4.0 Hz, 4H), 7.93 (t, *J*_1_ = 4.0 Hz, *J*_1_ = 8.0 Hz, 2H); ^13^C NMR (100 MHz, DMSO-*d*_6_): 161.19, 135.86, 132.93, 130.22, 128.03, 119.54 ppm.

### General Procedure for the Compound 3


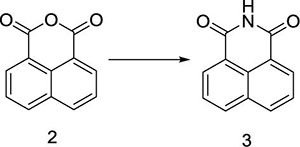


In a reaction flask, 1,8-naphthalic anhydride (Compound **2**, 50 g, 0.25 mol) was added to 1,000 mL of saturated ammonia water, and the mixture was stirred at room temperature for 10 min to obtain a yellow mixed liquid, which was slowly heated to 70°C. At this temperature, the reaction was carried out for 90 min. TLC was used to monitor the progress of the reaction. When the reaction was complete, heating was stopped. The flask was slowly cooled down to room temperature, solid precipitates appeared, and the reaction mixture was filtered. The filter cake was washed with 500 mL of water to neutralize it, and then dried at 60 °C to obtain 1,8-naphthalimide (Compound **3**,44 g), with a yield of 88%; ^1^H NMR (400 MHz, DMSO-*d*_6_): δ 8.42 (d, *J* = 4.0 Hz, 4H), 7.83 (t, *J*_1_ = 8.0 Hz, *J*_2_ = 4.0 Hz, 2H); ^13^C NMR (100 MHz, DMSO-*d*_6_): 164.56, 134.78, 132.03, 130.42, 127.53, 122.93ppm.

### General Procedure for the Preparation of Compound 4


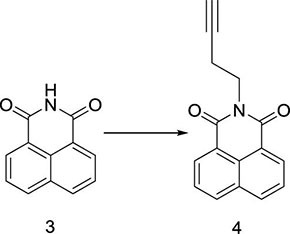


Compound **3** (10.00 g, 50.71 mmol) was added to a reaction flask and dissolved in N,N-dimethylformamide (DMF) (150 mL), and 4-bromo-1-butyne (7.42 g, 55.78 mmol) and potassium carbonate (21.02 g, 152.13 mmol) were added successively. Under nitrogen protection, the reaction was carried out at 100 °C overnight, and TLC was used to monitor the progress of the reaction. When the reaction was completed, the reaction mixture was filtered while it was hot, the filtrate was cooled at room temperature, and solid precipitates appeared. The filter cake was washed with a small amount of DMF and dried to obtain a white solid Compound **4** (9.65 g, 38.71 mmol), with a yield of 76.3%; ^1^H NMR (600 MHz, DMSO-d_6_) δ 8.50 (d, *J* = 7.2 Hz, 2H), 8.46 (d, *J* = 7.8 Hz, 2H), 7.86 (t, *J* = 7.8 Hz, 2H), 4.45 (t, *J* = 7.2 Hz, 2H), 3.18 (t, *J* = 7.2 Hz, 2H), 2.89 (s, 1H). ^13^C NMR (150 MHz, DMSO-d_6_) δ 163.85, 143.96, 134.65, 131.68, 128.63, 127.84, 124.26, 80.95, 56.53, 44.18, 24.92.

### General Procedure for the Preparation of Compound 5a–5o


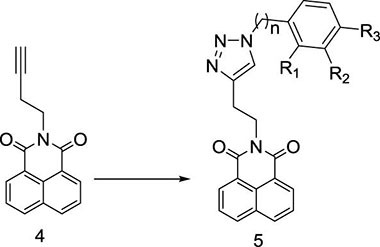


Aryl-azido (1.2 mmol) and compound **4** (1.0 mmol) were added to 15 mL mixed solvent (water/*tert*-butanol = 2:1). The reaction was catalyzed with CuI (0.1 mmol) at 80 °C. After completion of the reaction (monitored by TLC), the mixture was extracted with dichloromethane (15 mL × 3). The combined organic phase was washed successively with water and brine, dried over sodium sulfate and concentrated *in vacuo*. The residue was purified by through column chromatography (CH_2_Cl_2_/MeOH) to obtain the desired compound **5** as a crystalline powder.

#### 2-(2-(1-(2-fluorophenyl)-1H-1,2,3-triazol-4-yl)ethyl)-1H-benzo[de]isoquinoline-1,3(2H)-dione(5a)

^1^H NMR (600 MHz, DMSO-d_6_) δ 8.50 (d, *J* = 7.2 Hz, 2H), 8.49 (d, *J* = 1.8 Hz, 1H), 8.47 (d, *J* = 7.8 Hz, 2H), 7.88 (t, *J* = 7.8 Hz, 2H), 7.80 (td, *J*_1_ = 7.8 Hz, *J*_2_ = 1.2Hz, 1H), 7.61–7.58 (m, 1H), 7.57–7.53 (m, 1H), 7.43 (t, *J* = 8.4 Hz, 1H), 4.40 (t, *J* = 7.2 Hz, 2H), 3.14 (t, *J* = 7.8 Hz, 2H). ^13^C NMR (150 MHz, DMSO-d_6_) δ 163.88, 144.94, 134.83, 131.80, 131.55, 131.49, 131.21, 127.90, 127.71, 126.28, 126.00, 124.60, 124.57, 122.56, 117.68, 117.55, 24.00.

#### 2-(2-(1-(3-fluorophenyl)-1H-1,2,3-triazol-4-yl)ethyl)-1H-benzo[de]isoquinoline-1,3(2H)-dione (5b)

ield:69.5%; ^1^H NMR (400 MHz, DMSO-d_6_) δ 8.78 (s, 1H), 8.48 (dd, *J* = 7.2, 1.2 Hz, 2H), 8.45 (dd, *J*_1_ = 8.0 Hz, *J*_2_ = 1.2Hz, 2H), 7.88–7.84 (m, 2H), 7.81–7.74 (m, 2H), 7.66–7.59 (m, 1H), 7.35–7.28 (m, 1H), 4.38 (t, *J* = 8.0 Hz, 2H), 3.11 (t, *J* = 7.6 Hz, 2H). ^13^C NMR (100 MHz, DMSO-d_6_) δ 163.86, 161.68, 145.63, 134.81, 132.33, 132.24, 131.78, 131.19, 127.88, 127.67, 122.52, 121.49, 116.14, 115.68, 115.47, 107.82, 107.55, 39.70, 24.10.

#### 2-(2-(1-(4-fluorophenyl)-1H-1,2,3-triazol-4-yl)ethyl)-1H-benzo[de]isoquinoline-1,3(2H)-dione (5c)

ield: 35.9%; ^1^H NMR (400 MHz, DMSO-d_6_) δ 8.70 (s, 1H), 8.48 (dd, *J*_1_ = 7.2 Hz, *J*_2_ = 1.2 Hz, 2H), 8.45 (dd, *J*_1_ = 8.4 Hz, *J*_2_ = 1.2 Hz, 2H), 7.93–7.88 (m, 2H), 7.86 (m, 2H), 7.43 (t, *J* = 8.8 Hz, 2H), 4.38 (t, *J* = 7.6 Hz, 2H), 3.14–3.07 (t, *J* = 7.6 Hz, 2H). ^13^C NMR (100 MHz, DMSO-d_6_) δ 163.89, 145.47, 134.81, 133.79, 131.77, 131.22, 127.71, 122.65, 122.61, 122.57, 122.53, 121.55, 117.27, 117.07, 24.13.

#### 2-(2-(1-(2-bromophenyl)-1H-1,2,3-triazol-4-yl)ethyl)-1H-benzo[de]isoquinoline-1,3(2H)-dione (5d)

ield:36.1%; ^1^H NMR (600 MHz, DMSO-d_6_) δ 8.50 (dd, *J*_1_ = 7.2 Hz, *J*_2_ = 1.2Hz, 2H), 8.48–8.45 (m, 2H), 8.37 (s, 1H), 7.90–7.86 (m, 3H), 7.62–7.59 (m, 1H), 7.58 (dd, *J*_1_ = 7.8 Hz, *J*_2_ = 1.8Hz, 1H), 7.54–7.52 (m, 1H), 4.41 (t, *J* = 7.2 Hz, 2H), 3.14 (t, *J* = 7.2 Hz, 2H). ^13^C NMR (150 MHz, DMSO-d_6_) δ 163.84, 144.16, 136.84, 134.82, 134.02, 132.27, 131.80, 131.23, 129.38, 129.09, 127.91, 127.70, 125.30, 122.59, 119.28, 39.65, 23.95.

#### 2-(2-(1-(4-bromophenyl)-1H-1,2,3-triazol-4-yl)ethyl)-1H-benzo[de]isoquinoline-1,3(2H)-dione (5e)

ield29.2%; ^1^H NMR (400 MHz, DMSO-d_6_) δ 8.76 (s, 1H), 8.49 (d, *J* = 7.2 Hz, 2H), 8.46 (d, *J* = 8.4 Hz, 2H), 7.89–7.83 (m, 4H), 7.78 (d, *J* = 8.8 Hz, 2H), 4.38 (d, *J* = 7.6 Hz, 2H), 3.11 (d, *J* = 7.6 Hz, 2H). ^13^C NMR (100 MHz, DMSO-d_6_) δ 163.91, 145.65, 136.38, 134.83, 133.22, 131.79, 131.20, 127.89, 127.69, 122.53, 122.20, 122.17, 121.48, 121.39, 24.11.

#### 2-(2-(1-(2-chlorophenyl)-1H-1,2,3-triazol-4-yl)ethyl)-1H-benzo[de]isoquinoline-1,3(2H)-dione (5f)

Yield17.4%; ^1^H NMR (600 MHz, DMSO-d_6_) δ 8.54 (d, *J* = 7.2 Hz, 2H), 8.51 (d, *J* = 8.0 Hz, 2H), 8.44 (s, 1H), 7.93 (t, *J* = 7.8 Hz, 2H), 7.78 (d, *J* = 8.4 Hz, 1H), 7.68–7.64 (m, 2H), 7.62 (t, *J* = 7.8 Hz, 1H), 4.46 (t, *J* = 7.2 Hz, 2H), 3.20 (t, *J* = 7.2 Hz, 2H). ^13^C NMR (150 MHz, DMSO-d_6_) δ 163.84, 144.27, 135.16, 134.81, 131.92, 131.79, 131.21, 130.95, 128.96, 128.87, 128.76, 127.90, 127.68, 125.30, 122.57, 39.65, 23.97.

#### 2-(2-(1-(2-methoxyphenyl)-1H-1,2,3-triazol-4-yl)ethyl)-1H-benzo[de]isoquinoline-1,3(2H)-dione (5g)

ield7.5%; ^1^H NMR (600 MHz, DMSO-d_6_) δ 8.51 (d, *J* = 7.2 Hz, 2H), 8.47 (d, *J* = 8.4 Hz, 2H), 8.31 (s, 1H), 7.89 (t, *J* = 7.8 Hz, 2H), 7.58 (dd, *J*_1_ = 7.8 Hz, *J*_2_ = 1.8 Hz, 1H), 7.53–7.48 (m, 1H), 7.30 (d, *J* = 8.3 Hz, 1H), 7.13 (t, *J* = 7.6 Hz, 1H), 4.40 (t, *J* = 7.8 Hz, 2H), 3.78 (s, 3H), 3.12 (t, *J* = 7.2 Hz, 2H). ^13^C NMR (151 MHz, DMSO-d_6_) δ 163.87, 152.00, 144.01, 134.82, 131.80, 131.22, 130.95, 127.90, 127.71, 126.34, 126.07, 124.91, 122.58, 121.32, 113.47, 56.48, 24.05.

#### 2-(2-(1-(4-nitrophenyl)-1H-1,2,3-triazol-4-yl)ethyl)-1H-benzo[de]isoquinoline-1,3(2H)-dione (5h)

ield30.5%; ^1^H NMR (600 MHz, DMSO-d_6_) δ 8.97 (s, 1H), 8.52 (d, *J* = 7.2 Hz, 2H), 8.49 (d, *J* = 8.4 Hz, 2H), 8.45 (d, *J* = 8.4 Hz, 2H), 8.20 (d, *J* = 8.4 Hz, 2H), 7.90 (t, *J* = 7.8 Hz, 2H), 4.42 (t, *J* = 7.8 Hz, 2H), 3.15 (t, *J* = 7.8 Hz, 2H). ^13^C NMR (150 MHz, DMSO-d_6_) δ 163.94, 147.01, 146.19, 141.42, 134.88, 131.84, 131.26, 127.96, 127.74, 126.10, 122.60, 121.84, 120.77, 40.53, 24.09.

#### 2-(2-(1-benzyl-1H-1,2,3-triazol-4-yl)ethyl)-1H-benzo[de]isoquinoline-1,3(2H)-dione (5i)

^1^H NMR (600 MHz, DMSO-d_6_) δ 8.46 (t, *J* = 7.2 Hz, 4H), 8.02 (s, 1H), 7.87 (t, *J* = 7.8 Hz, 2H), 7.34 (t, *J* = 7.2 Hz, 2H), 7.31 (t, *J* = 6.6 Hz, 1H), 7.21 (d, *J* = 6.6 Hz, 2H), 5.54 (s, 2H), 4.31 (t, *J* = 7.2 Hz, 2H), 3.03 (t, *J* = 7.2 Hz, 2H). ^13^C NMR (150 MHz, DMSO-d_6_) δ 163.81, 136.77, 134.80, 131.78, 131.20, 129.13, 128.39, 128.00, 127.85, 127.69, 123.38, 122.50, 53.04, 24.06.

#### 2-(2-(1-(2-fluorobenzyl)-1H-1,2,3-triazol-4-yl)ethyl)-1H-benzo[de]isoquinoline-1,3(2H)-dione (5j)

^1^H NMR (600 MHz, DMSO-d_6_) δ 8.49–8.41 (m, 4H), 8.00 (s, 1H), 7.86 (t, *J* = 7.2 Hz, 2H), 7.43–7.38 (m, 1H), 7.23 (t, *J* = 9.0 Hz, 1H), 7.20–7.17 (m, 2H), 5.60 (s, 2H), 4.31 (t, *J* = 7.2 Hz, 2H), 3.03 (t, *J* = 7.2 Hz, 2H). ^13^C NMR (150 MHz, DMSO-d_6_) δ 163.79, 134.77, 131.76, 131.17, 130.96, 130.91, 130.66, 130.64, 127.66, 125.24, 125.22, 123.63, 123.53, 123.43, 122.47, 116.03, 115.89, 47.11, 24.03.

#### 2-(2-(1-(2-bromobenzyl)-1H-1,2,3-triazol-4-yl)ethyl)-1H-benzo[de]isoquinoline-1,3(2H)-dione (5k)

^1^H NMR (600 MHz, DMSO-d_6_) δ 8.46 (t, *J* = 7.8 Hz, 4H), 7.99 (s, 1H), 7.87 (t, *J* = 7.8 Hz, 2H), 7.65 (d, *J* = 7.8 Hz, 1H), 7.37 (t, *J* = 7.2 Hz, 1H), 7.29 (t, *J* = 7.8 Hz, 1H), 6.95 (d, *J* = 7.8 Hz, 1H), 5.60 (s, 2H), 4.33 (t, *J* = 7.2 Hz, 2H), 3.05 (t, *J* = 7.2 Hz, 2H). ^13^C NMR (150 MHz, DMSO-d_6_) δ 163.84, 144.40, 135.67, 134.81, 133.21, 131.78, 131.21, 130.61, 130.15, 128.67, 127.86, 127.68, 123.74, 122.89, 122.50, 53.16, 39.80, 24.02.

#### 2-(2-(1-(3-bromobenzyl)-1H-1,2,3-triazol-4-yl)ethyl)-1H-benzo[de]isoquinoline-1,3(2H)-dione (5l)

^1^H NMR (600 MHz, DMSO-d_6_) δ 8.46 (t, *J* = 7.2 Hz, 4H), 8.05 (s, 1H), 7.86 (t, *J* = 7.8 Hz, 2H), 7.53 (d, *J* = 8.4 Hz, 1H), 7.49 (s, 1H), 7.31 (t, *J* = 7.8 Hz, 1H), 7.20 (d, *J* = 7.8 Hz, 1H), 5.55 (s, 2H), 4.31 (t, *J* = 7.2 Hz, 2H), 3.03 (t, *J* = 7.2 Hz, 2H). ^13^C NMR (150 MHz, DMSO-d_6_) δ 163.81, 144.61, 139.39, 134.79, 131.78, 131.34, 131.18, 130.89, 127.85, 127.67, 127.15, 123.47, 122.50, 122.28, 52.22, 39.81, 24.06.

#### 2-(2-(1-(2-methylbenzyl)-1H-1,2,3-triazol-4-yl)ethyl)-1H-benzo[de]isoquinoline-1,3(2H)-dione (5m)

^1^H NMR (600 MHz, DMSO-d_6_) δ 8.46 (d, *J* = 1.8 Hz, 2H), 8.45 (d, *J* = 3.0 Hz, 2H), 7.87 (t, *J* = 7.8 Hz, 3H), 7.22 (t, *J* = 7.2 Hz, 1H), 7.18 (d, *J* = 7.2 Hz, 1H), 7.14 (t, *J* = 7.2 Hz, 1H), 6.94 (d, *J* = 7.2 Hz, 1H), 5.52 (s, 2H), 4.31 (t, *J* = 7.2 Hz, 2H), 3.03 (t, *J* = 7.2 Hz, 2H), 2.24 (s, 3H). ^13^C NMR (151 MHz, DMSO-d_6_) δ 163.81, 144.36, 136.55, 134.78, 131.78, 131.18, 130.73, 128.64, 128.55, 127.84, 127.67, 126.63, 123.28, 122.49, 51.27, 39.79, 24.02, 19.02.

#### 2-(2-(1-(4-methylbenzyl)-1H-1,2,3-triazol-4-yl)ethyl)-1H-benzo[de]isoquinoline-1,3(2H)-dione (5n)

^1^H NMR (600 MHz, DMSO-d_6_) δ 8.45 (dd, *J*_1_ = 7.2 Hz, *J*_2_ = 4.8 Hz, 4H), 7.97 (s, 1H), 7.87 (t, *J* = 7.8 Hz, 2H), 7.14–7.10 (m, 4H), 5.47 (s, 2H), 4.30 (t, *J* = 7.2 Hz, 2H), 3.01 (t, *J* = 7.2 Hz, 2H), 2.28 (s, 3H). ^13^C NMR (150 MHz, DMSO-d_6_) δ 163.79, 137.68, 134.78, 133.71, 131.77, 131.19, 129.65, 128.09, 127.84, 127.66, 123.12, 122.49, 52.87, 39.78, 24.05, 21.16.

#### 2-(2-(1-(3,4-dimethoxybenzyl)-1H-1,2,3-triazol-4-yl)ethyl)-1H-benzo[de]isoquinoline-1,3(2H)-dione (5o).

^1^H NMR (600 MHz, DMSO-d_6_) δ 8.67 (s, 1H), 8.50 (d, *J* = 7.2 Hz, 2H), 8.47 (d, *J* = 8.4 Hz, 2H), 7.88 (t, *J* = 7.8 Hz, 2H), 7.42 (d, *J* = 2.4 Hz, 1H), 7.37 (dd, *J*_1_ = 8.4 Hz, *J*_2_ = 2.4 Hz, 1H), 7.11 (d, *J* = 9.0 Hz, 1H), 4.39 (t, *J* = 7.8 Hz, 2H), 3.85 (s, 3H), 3.82 (s, 3H), 3.10 (t, *J* = 7.8 Hz, 2H). ^13^C NMR (150 MHz, DMSO-d_6_) δ 163.88, 149.76, 149.14, 145.12, 134.83, 131.80, 131.21, 130.69, 127.91, 127.70, 122.58, 121.36, 112.49, 112.29, 104.88, 56.31, 56.27, 39.79, 24.15.

### MTT Assay for Cell Proliferation and Cytotoxicity

Cells (approximately 3,000–5,000 cells/well) were seeded in 96-well plates and cultured for 16 h until the cells were adherent. The concentration of test compounds was varied between 2, 4, 8, 16, and 32 μM, 0.1% DMSO was added to cells as control five multiple wells. Plates were cultured at 37 °C in 5% CO_2_ environment for 72 h. After treatment, 10 μL of MTT (5 mg/mL) was added to each well and the plates were incubated at 37 °C and 5% CO_2_ for 4 h. The reaction mixture containing 10% SDS and 0.1 mM of HCL was then added to each well before plates were incubated at 37 °C for another 4 h. The absorbance was measured on a microplate reader at a wavelength of 570 nm (Wang et al., 2020).

## Data Availability Statement

The original contributions presented in the study are included in the article/Supplementary Material, further inquiries can be directed to the corresponding author/s.

## Author Contributions

L-FM and WL conceived the study, designed the experiments, and supervised all research. Z-JX prepared the draft of the manuscript. Z-JX and Y-JZ synthesized all compounds. J-HW carried out the experiments and analyzed the data. G-QX contributed to the preliminary activity test and article collation of this paper. All authors reviewed the manuscript.

## Conflict of Interest

The authors declare that the research was conducted in the absence of any commercial or financial relationships that could be construed as a potential conflict of interest.
