# Supplementary material for: The Synthesis and Antitumor Activity of 1,8-Naphthalimide Derivatives Linked 1,2,3-Triazole
Source: Front Bioeng Biotechnol. 2021 Apr 13;9:662432. doi: 10.3389/fbioe.2021.662432 (PMC8076741; doi:10.3389/fbioe.2021.662432)

*Supporting information*

**The synthesis and antitumor activity of 1,8-naphthalimide derivatives linked 1,2,3-triazole**

Zhong-jie Xu^a^, Ying-jie Zhou^b^, Jia-hao Wang^b^, Long-fei Mao^b^, Wei Li^b^*

^a^Life Science Department, Xinxiang Medical College, Xinxiang 453003, China

^b^School of Chemistry and Chemical Engineering, Henan Normal University, Henan Engineering Research Center of Chiral Hydroxyl Pharmaceutical, Xinxiang 453007, China.

Content

[Figure S1. ^1^H NMR spectrum (600 MHz, DMSO-d_6_) of compound 5a 1](#_Toc57386835)

[Figure S2. ^1^H NMR spectrum (600 MHz, DMSO-d_6_) of compound 5b 2](#_Toc57386836)

[Figure S3. ^1^H NMR spectrum (600 MHz, DMSO-d_6_) of compound 5c 3](#_Toc57386837)

[Figure S4. ^1^H NMR spectrum (600 MHz, DMSO-d_6_) of compound 5d 4](#_Toc57386838)

[Figure S5. ^1^H NMR spectrum (600 MHz, DMSO-d_6_) of compound 5e 5](#_Toc57386839)

[Figure S6. ^1^H NMR spectrum (600 MHz, DMSO-d_6_) of compound 5f 6](#_Toc57386840)

[Figure S7. ^1^H NMR spectrum (600 MHz, DMSO-d_6_) of compound 5g 7](#_Toc57386841)

[Figure S8. ^1^H NMR spectrum (600 MHz, DMSO-d_6_) of compound 5h 8](#_Toc57386842)

[Figure S9. ^1^H NMR spectrum (600 MHz, DMSO-d_6_) of compound 5i 9](#_Toc57386843)

[Figure S10. ^1^H NMR spectrum (600 MHz, DMSO-d_6_) of compound 5j 10](#_Toc57386844)

[Figure S11. ^1^H NMR spectrum (600 MHz, DMSO-d_6_) of compound 5k 11](#_Toc57386845)

[Figure S12. ^1^H NMR spectrum (600 MHz, DMSO-d_6_) of compound 5l 12](#_Toc57386846)

[Figure S13. ^1^H NMR spectrum (600 MHz, DMSO-d_6_) of compound 5m 13](#_Toc57386847)

[Figure S14. ^1^H NMR spectrum (600 MHz, DMSO-d_6_) of compound 5n 14](#_Toc57386848)

[Figure S15. ^1^H NMR spectrum (600 MHz, DMSO-d_6_) of compound 5o 15](#_Toc57386849)

# Figure S1. ^1^H NMR spectrum (600MHz, DMSO-d_6_) of compound 5a


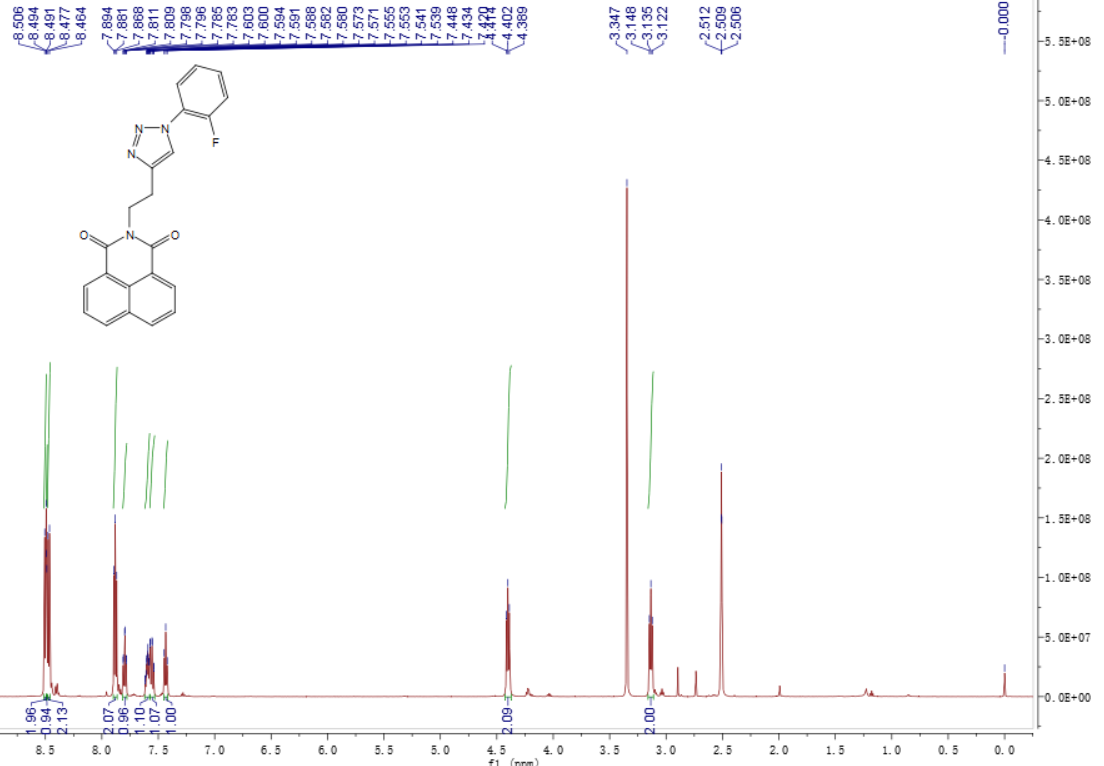


# Figure S2. ^1^H NMR spectrum (600MHz, DMSO-d_6_) of compound 5b


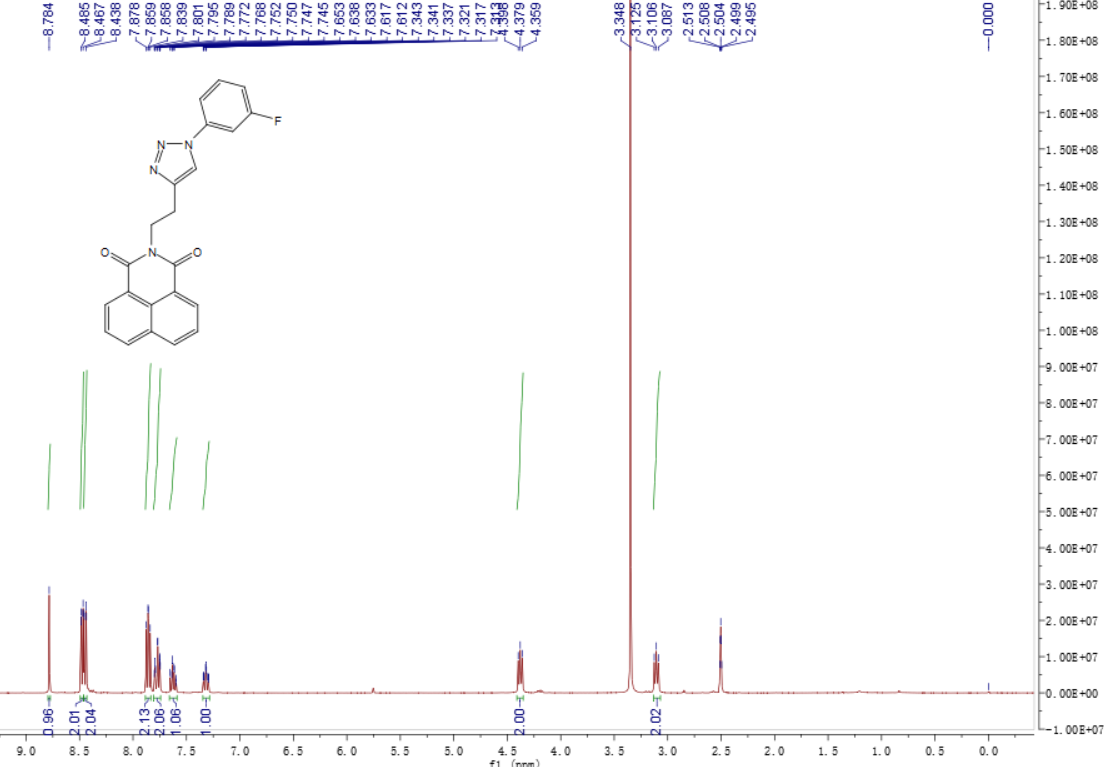


# Figure S3. ^1^H NMR spectrum (600MHz, DMSO-d_6_) of compound 5c


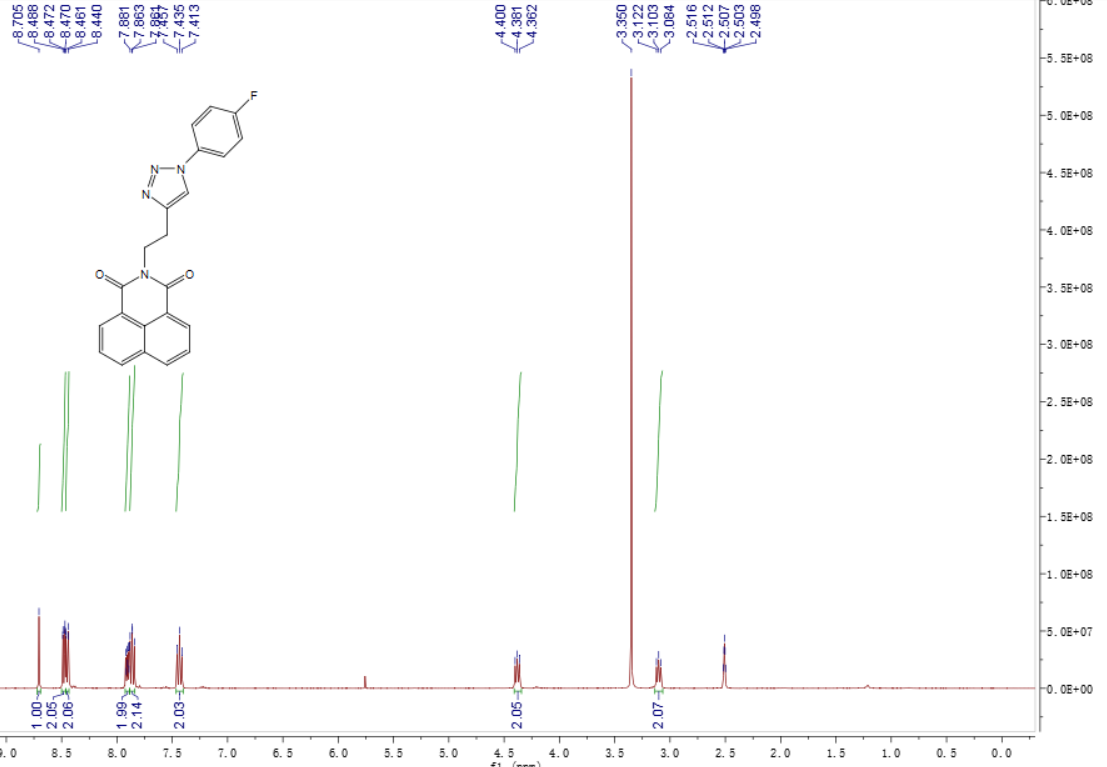


# Figure S4. ^1^H NMR spectrum (600MHz, DMSO-d_6_) of compound 5d


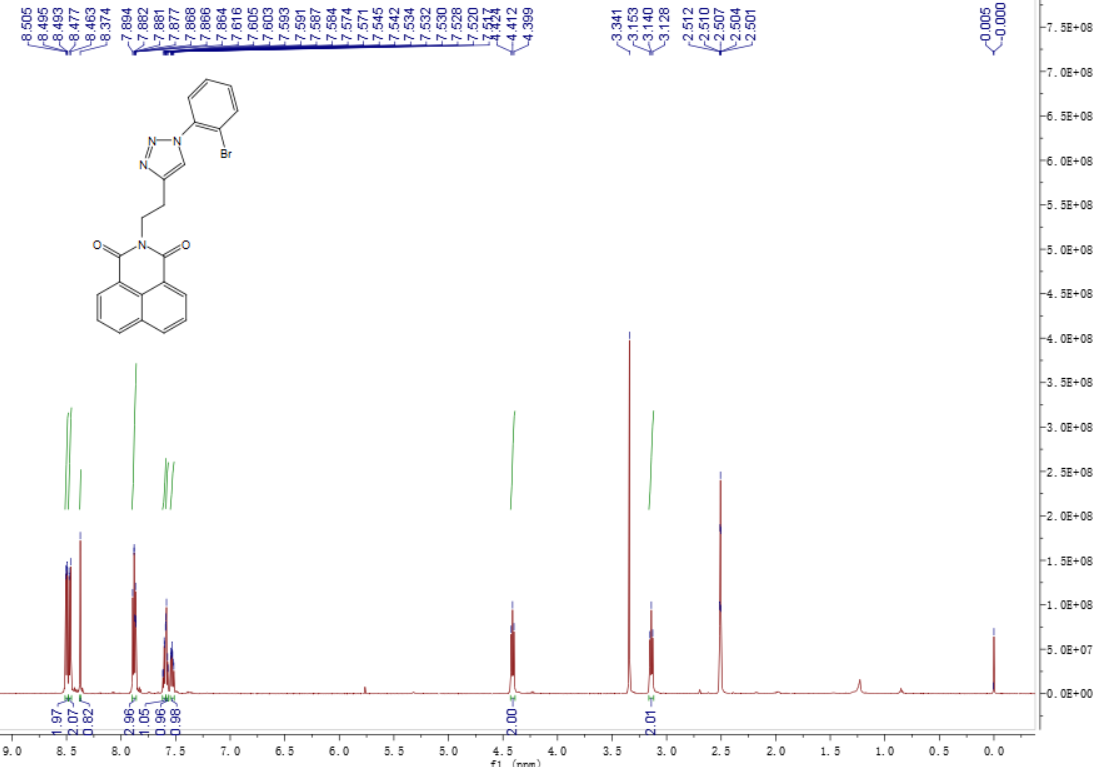


# Figure S5. ^1^H NMR spectrum (600MHz, DMSO-d_6_) of compound 5e


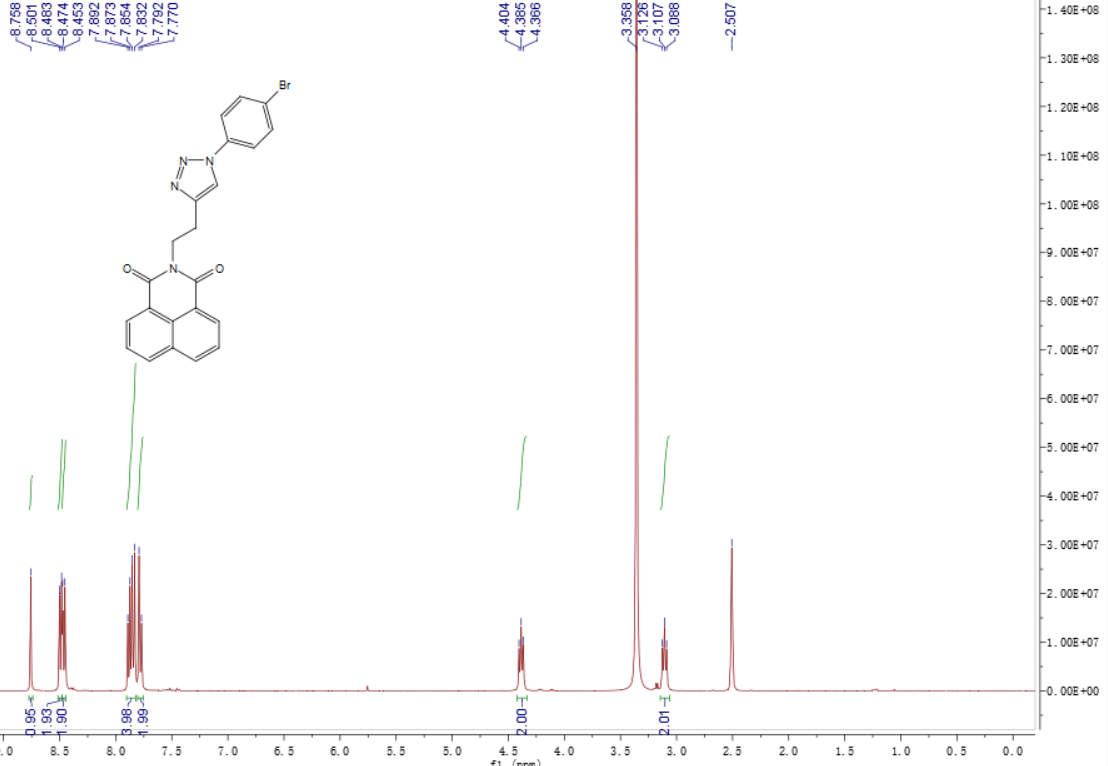


# Figure S6. ^1^H NMR spectrum (600MHz, DMSO-d_6_) of compound 5f


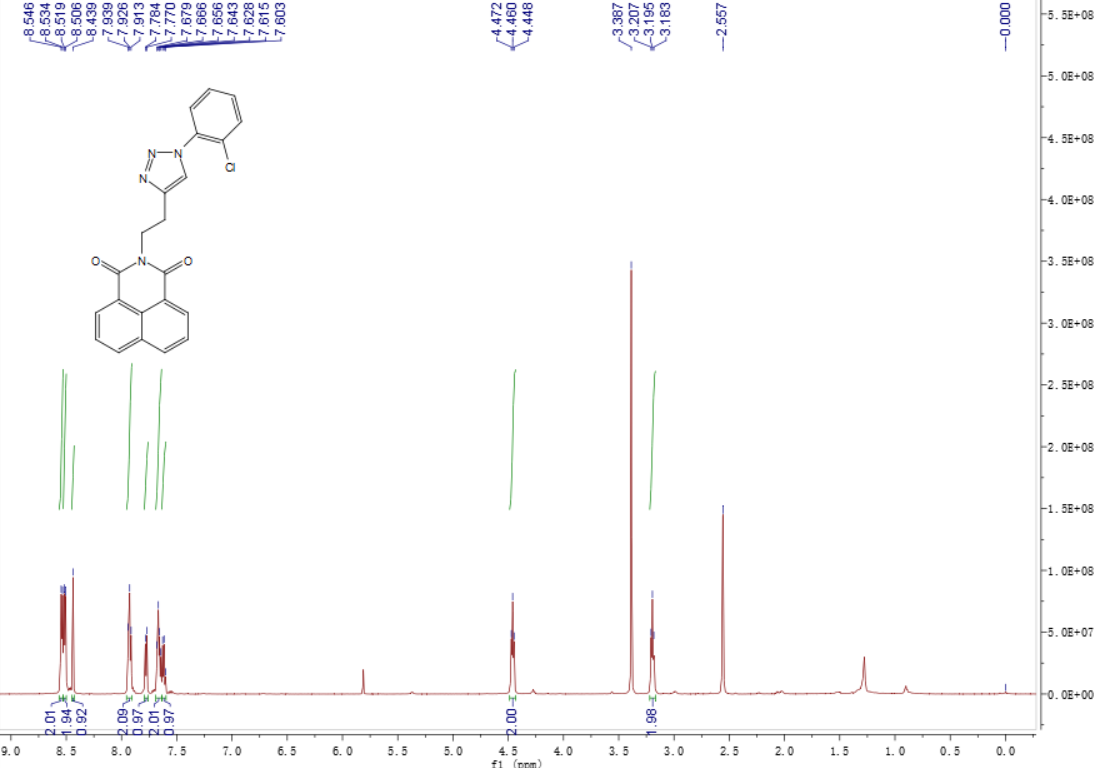


# Figure S7. ^1^H NMR spectrum (600MHz, DMSO-d_6_) of compound 5g


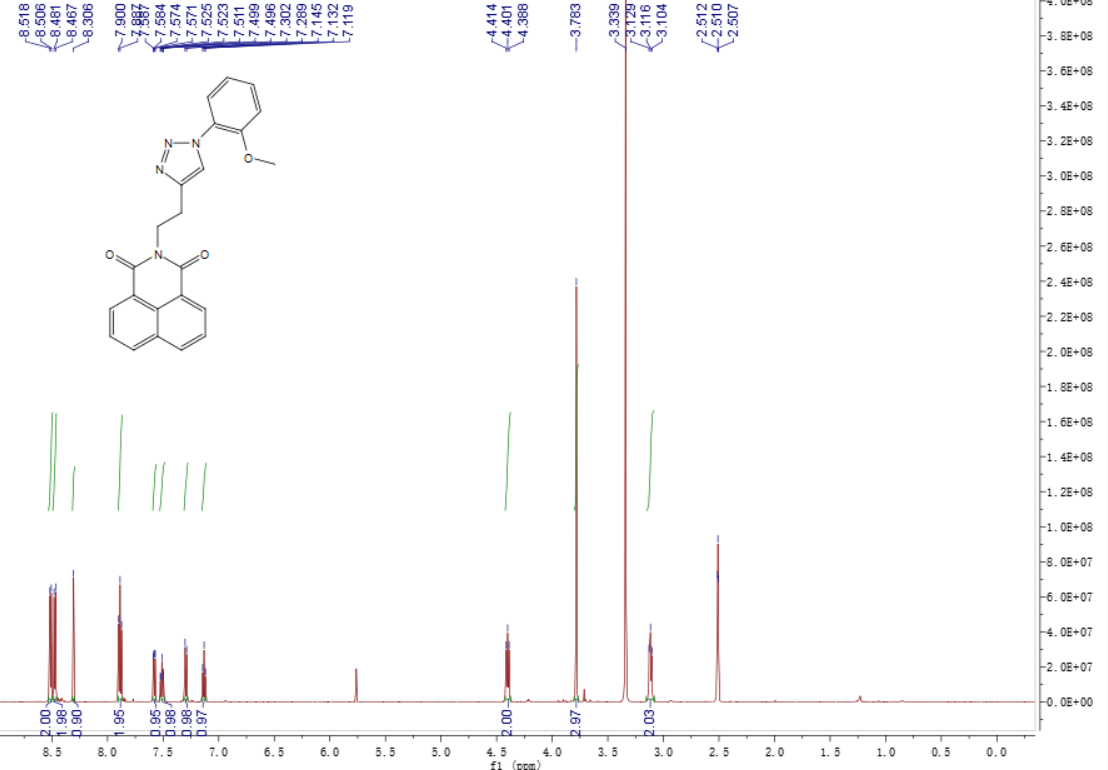


# Figure S8. ^1^H NMR spectrum (600MHz, DMSO-d_6_) of compound 5h


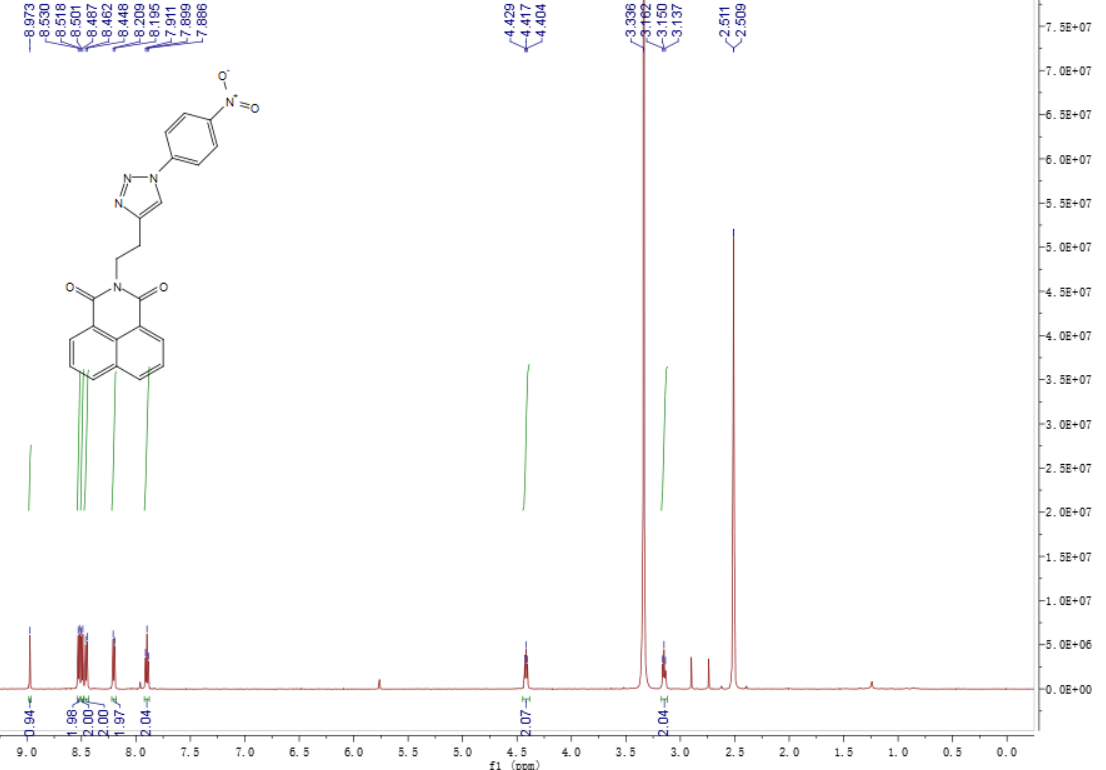


# Figure S9. ^1^H NMR spectrum (600MHz, DMSO-d_6_) of compound 5i


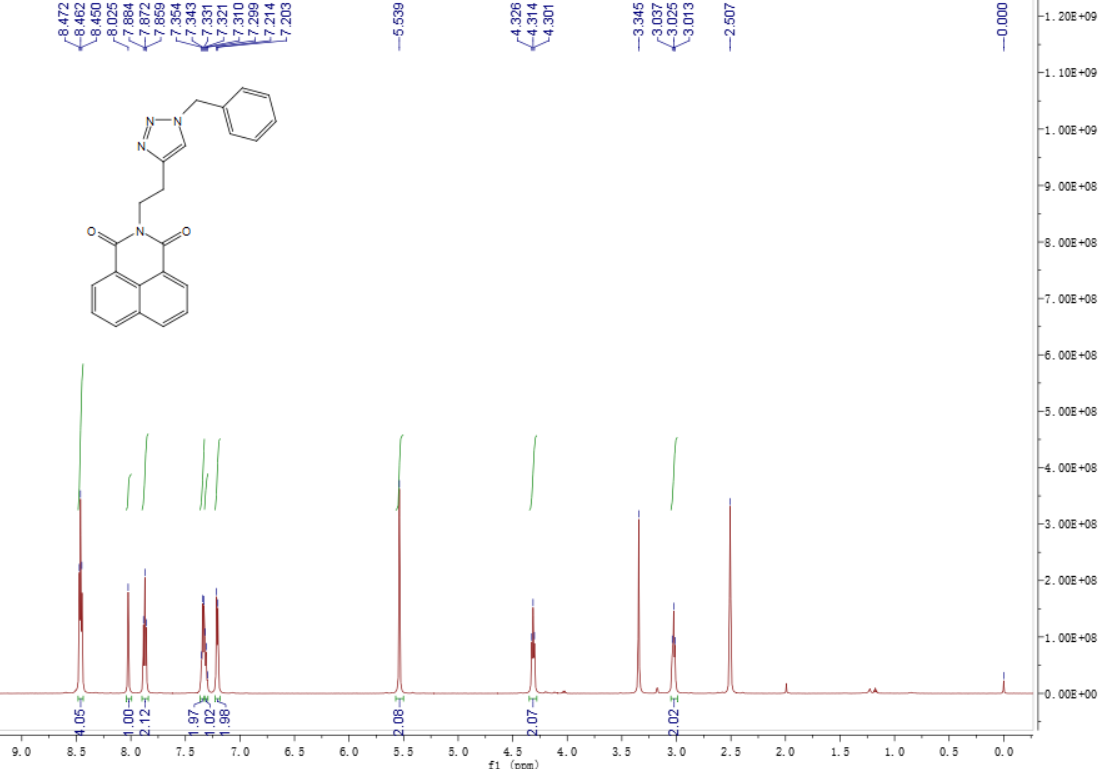


# Figure S10. ^1^H NMR spectrum (600MHz, DMSO-d_6_) of compound 5j


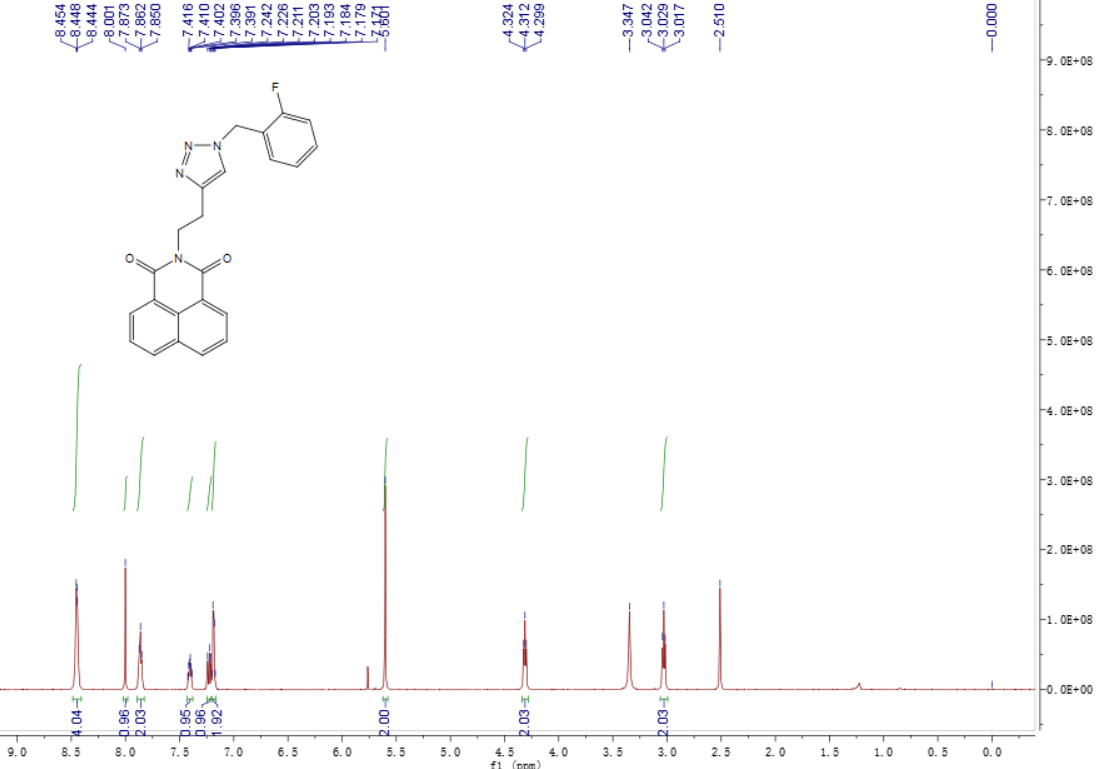


# Figure S11. ^1^H NMR spectrum (600MHz, DMSO-d_6_) of compound 5k


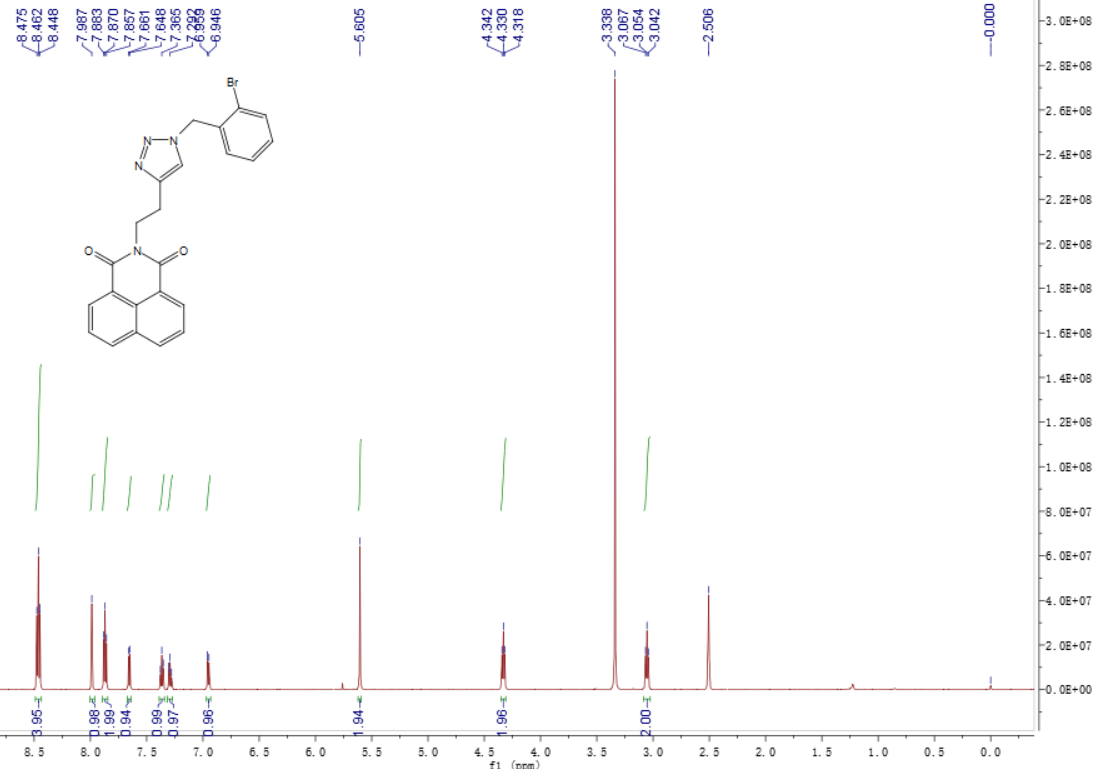


# Figure S12. ^1^H NMR spectrum (600MHz, DMSO-d_6_) of compound 5l


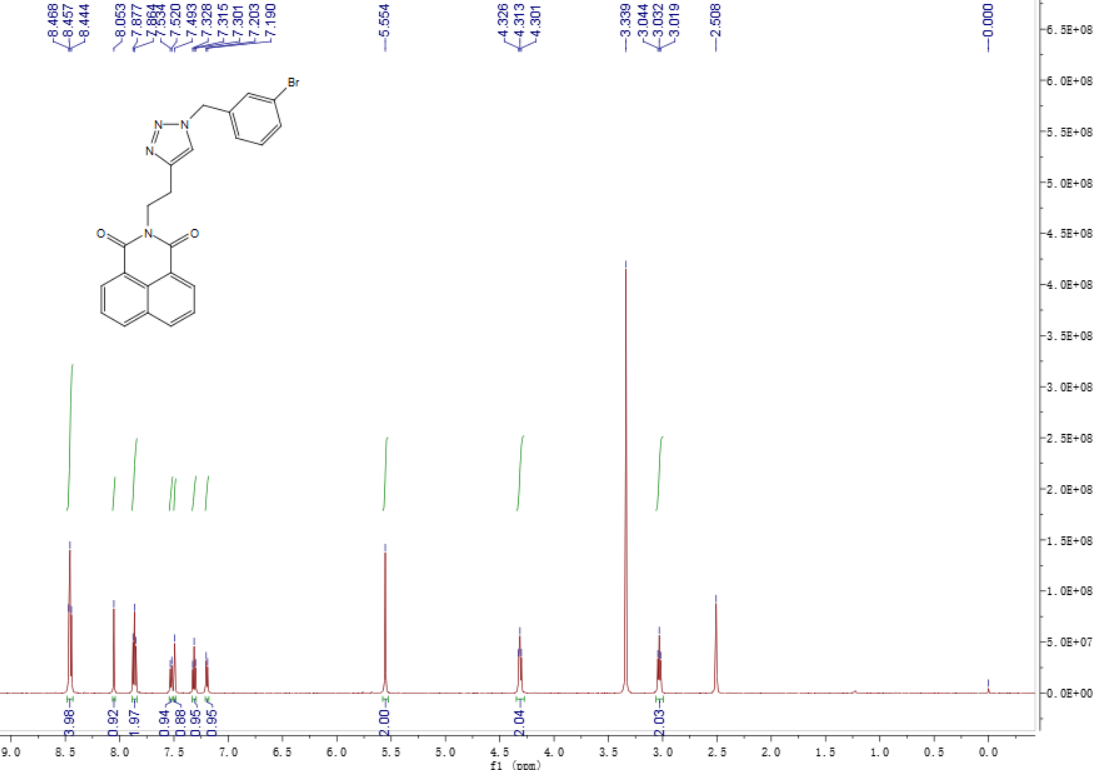


# Figure S13. ^1^H NMR spectrum (600MHz, DMSO-d_6_) of compound 5m


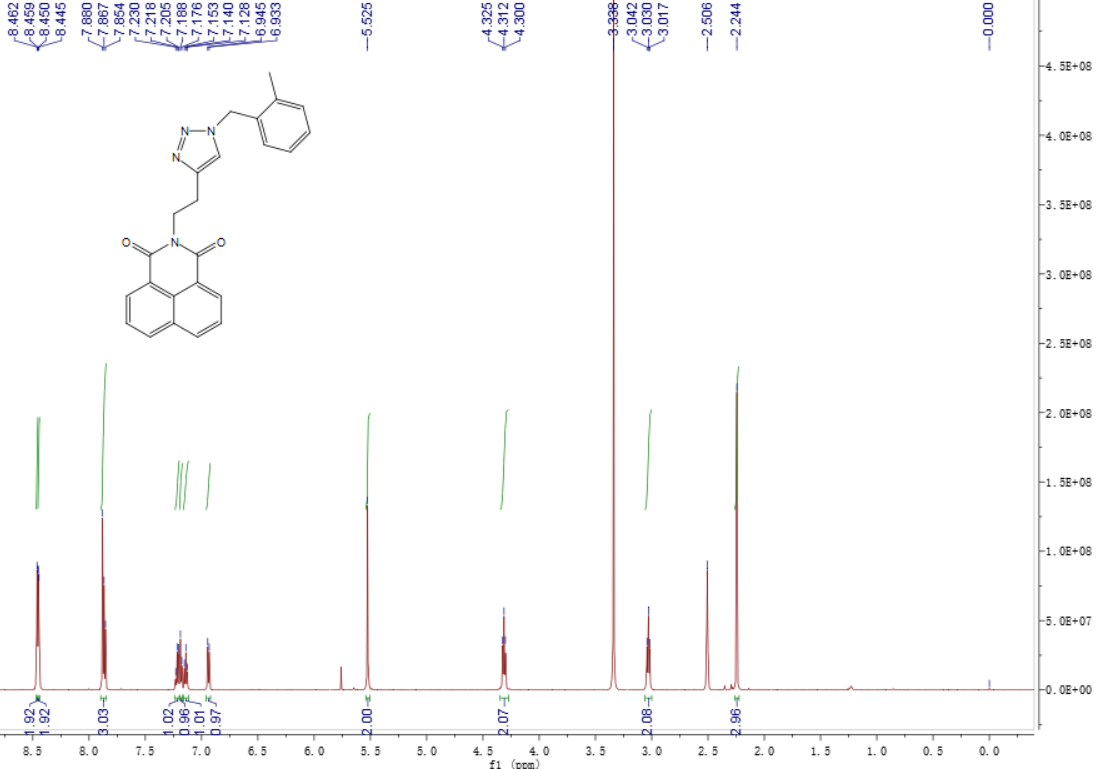


# Figure S14. ^1^H NMR spectrum (600MHz, DMSO-d_6_) of compound 5n


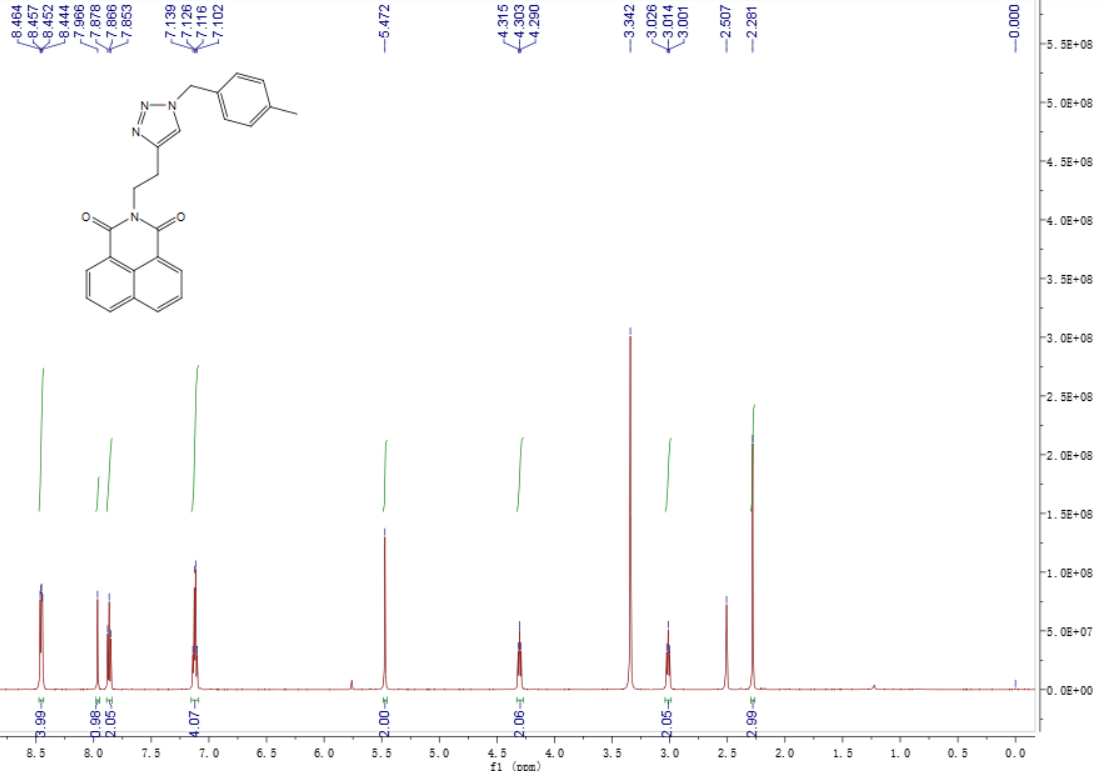


# Figure S15. ^1^H NMR spectrum (600MHz, DMSO-d_6_) of compound 5o


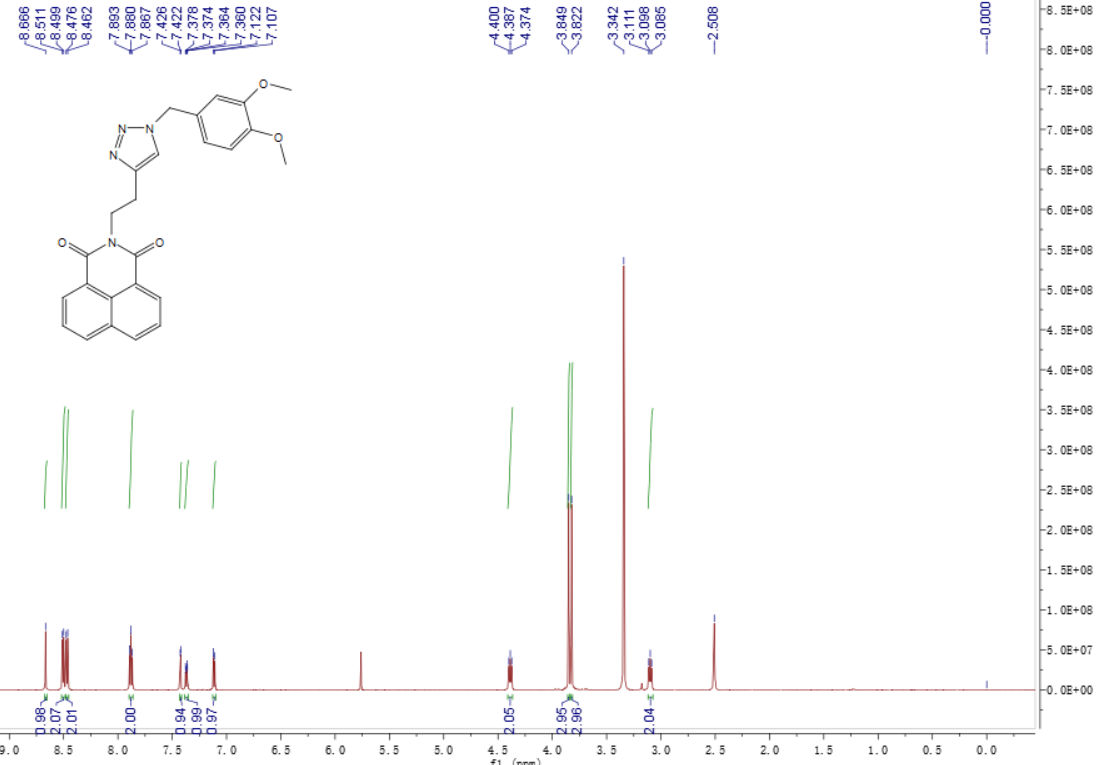

Supplement: Supplementary file 1 [file Data_Sheet_1.docx]
